# Relation between the dash score and radiographic evaluation of the wrist in patients with wrist fracture

**DOI:** 10.1186/s12891-024-07307-2

**Published:** 2024-03-15

**Authors:** Anthony Hassoun, Rami Haroun, Fadi Hoyek, Jean Claude Lahoud, Charbel Tawk, Majd El Hajj Moussa, Rita Khalil, Joseph Mandour, Boutros El Tannoury

**Affiliations:** 1 Department of Orthopedic Surgery, CHU Notre Dame Des Secours, Holy Spirit University of Kaslik – School of Medicine and Medical Sciences, Jbeil, Lebanon; 2 Department of Orthopedics at Notre Dame des Secours University Hospital, Jbeil, Lebanon

**Keywords:** DASH score, Wrist fracture, Distal radius fracture, Ulnar variance, Radial height, Radial inclination, Volar tilt

## Abstract

Traditionally, the assessment of distal radius fracture outcomes has been based on radiological measurements and self-evaluation scores. However, there is uncertainty regarding how accurately these measurements reflect the patient's perception of their outcome. In this study, we examined the correlation between radiological measurements and patient-perceived outcomes using the Disabilities of the Arm, Shoulder, and Hand outcome (DASH) score. 140 individuals who had recovered from a distal radius fracture. and had been treated with DVR, Kapandji, percutaneous pinning or closed reduction were included in the study. The retrospective assessment included 78 females and 62 males, with a mean DASH score of 3.54 points.

Except for the ulnar variance, the study found little to no significant association between the DASH score and the final radiological measurement.

In summary, the DASH score did not always indicate that a superior radiological result translated into a better patient-perceived outcome.

## Introduction

### Background

Distal radial fractures are a common injury in both adults and children, that is often caused by low-energy trauma such as hyperextension or bending forces, or high-energy trauma from compression forces [1–5].

Conventional posteroanterior and lateral x-ray perspectives are usually used to evaluate radial length (12 mm on average), radial inclination (23° on average), ulnar variance (-2 mm to 2 mm), and lateral x-ray perspectives to evaluate volar tilt (12° average). In clinical practice, it is essential to follow the guidelines for assessing radial length, radial inclination, volar tilt, and ulnar variation in wrist x-rays in order to guarantee proper diagnosis, treatment planning, progress tracking, and outcome prediction. A radial shortening greater than 4 mm was linked with wrist pain at a mean follow-up of 23 months. Following a mean of 22 months, there was a correlation between a worse DASH score and a loss of radial inclination of more than 10 degrees in healed distal radial fractures. Healthcare professionals can precisely assess the wrist joint's alignment and stability, choose the best course of action for treating wrist injuries or conditions, monitor the effectiveness of treatment by comparing measured results to established norms, and make more accurate prognostic assessments when these norms are deviated from. In general, following these recommendations improves the standard of treatment given to patients who have ailments or injuries related to the wrist [3, 4, 6–9].

There are various classifications for these fractures, Fernandez is a known classification that divides distal radial fractures into five categories based on radiographic features, fracture cause, and stress direction: Bending fractures as Type I, joint surface shearing fractures as Type II, joint surface compression fractures as Type III, radiocarpal fracture dislocations or avulsion fractures as Type IV, and high-velocity events associated with combination fractures are classified as Type V [3, 4, 10, 11].

The goal of treatment, whether conservative or surgical, is to restore alignment. Radiographic standards have been established for acceptable reduction. This includes radial shortening of less than 5 mm, radial inclination of greater than 15 degrees, volar tilt of 15 to 20 degrees, intra-articular step-off of the radiocarpal joint of less than 2 mm, and articular incongruity of the sigmoid notch of the distal radius of less than 2 mm [4]. Nonoperative treatments include plaster or splint therapy with or without closure reduction is typically used for undisplaced fractures [4, 12].

Operative treatments for distal radius fractures encompass various techniques tailored to the specific characteristics of the fracture. Percutaneous pinning is employed for unstable extra-articular or minimal articular fractures, as well as for intra-articular fractures. Bridging external fixation is indicated for cases of actual or anticipated instability in dorsally displaced extra-articular or minor articular fractures, open wounds, and severe articular fractures. Nonbridging external fixation is primarily used for distal radius fractures with actual or anticipated instability, including those extra-articular or with an articular extension, and undisplaced or reducible closed fractures. Volar locked plating, sharing similar indications to nonbridging external fixation, is suitable for unstable or potentially unstable distal radius fractures, where the distal fragment provides enough space for pins. It can also correct malunions through a corrective osteotomy and treat volar displacement fractures. Dorsal plating is employed in minimally articular or dorsally displaced extra-articular distal radius fractures. Each technique offers specific advantages and is chosen based on the individual characteristics and needs of the patient's fracture [3, 4, 13].

Treatment decisions for distal radial fractures are multifaceted and influenced by several factors, including age, psychological state, socioeconomic status, fracture type, and radiological parameters such as radial height, ulnar variance, radial inclination, and volar tilt.

Age influences treatment decisions in distal radial fractures with younger patients often undergoing surgical intervention to meet higher functional demands, while conservative management is favored in older and elderly patients, considering factors such as bone quality, comorbidities, and functional goals. Socioeconomic status impacts treatment decisions for distal radial fractures, with higher SES individuals often accessing specialized care and opting for surgical interventions due to better healthcare access and affordability. Lower SES individuals may face barriers to care, leading to reliance on conservative management and potentially delayed or limited access to surgical treatments. Fracture type profoundly influences the treatment approach for distal radial fractures. Extra-articular fractures typically respond well to conservative management, while intra-articular fractures often require surgical intervention to restore joint alignment and function. Displacement and stability guide the decision between conservative treatment or surgical fixation, with comminution and fragmentation presenting additional challenges that may necessitate meticulous surgical techniques for optimal outcomes.

Radiological parameters such as radial height, ulnar variance, radial inclination, and volar tilt significantly influence treatment decisions for distal radial fractures. Reduced radial height, positive ulnar variance, loss of radial inclination, and decreased volar tilt may necessitate surgical intervention to restore anatomical alignment and prevent long-term complications, while conservative management may be considered for mild deviations with low functional impact [14, 15].

Complications can arise from treatment and may include compartment syndrome (0.3%), nerve damage (2–8%), wrist stiffness (37.8%), nonunion (0.5%), malunion (0.3%), tendon rupture (1.3%) and tendon infections (1.5%) [16, 17].

The DASH score, a 30-item self-report questionnaire is a useful and reliable tool for evaluating physical function and disability in people with upper-extremity musculoskeletal conditions including distal radial fractures. It can help measure the level of disability and track changes in symptoms and function over time, providing valuable insights into the impact of these injuries on daily activities and quality of life [18].

### Objective

The aim of the study is to assess the reliability of the DASH score in patients with wrist fracture in our population and to study how the changes in the anatomical indexes of the wrist will affect the values of the DASH score.

## Material and methods

### Study design and patients

This study is a retrospective longitudinal cohort study that targeted patients with distal radius fractures who underwent operative or non-operative treatment at CHU-NDS between January 2017 and December 2022. Data analyzed in this study was obtained from the hospital archive after receiving approval from the CHU-NDS ethical committee. Inclusion criteria are patients with radial distal fractures who have underwent x-rays in our emergency department and have been treated in our hospital. Exclusion criteria are patients with psychological or physical impairments that would prevent them from properly completing the questionnaire. A total of 174 patients who met our criteria have been selected for the study. We contacted all patients to fill out the DASH score via phone call after explaining the goals and objectives of our study and obtaining their oral consent to participate. To protect patient privacy, data was gathered and separated from patient identity information. However, 34 patients (19.5%) did not answer our phone calls or refused to complete the questionnaire, leading to non-response bias. This bias occurs because individuals who choose not to respond may have different perspectives, experiences, or outcomes compared to those who do respond. Additionally, it causes information bias as non-responders may have missing data that could impact the completeness and accuracy of the study findings or clinical assessments. We enhanced outreach efforts by following up with non-responders through numerous contact attempts in an effort to encourage participation and lower non-response rates, mitigating the biases introduced by non-response.

Sociodemographic data collected for each patient included gender, age, and ethnicity.

Clinical data included the severity of trauma, any associated fractures of the ipsilateral arm, surgical method, and operating time.

### Dash score

The Arabic version of the DASH score was used and was filled between 6 months and up to 5 years post-surgery. It is a 30-item self-report questionnaire created to evaluate physical function and disability in people with diseases of the musculoskeletal system in the upper extremities. Patients with upper-extremity disabilities have demonstrated in previous studies responsiveness, reliability, and validity of the DASH questionnaire [19].

The questionnaire asks about the severity of each symptom, such as pain, tingling, activity-related pain, stiffness, and weakness (5 items). It also evaluate how the problem affects sleep, work, social activities, and one's self-image (4 items). Moreover, it asks about how difficult it is to perform different physical activities due to hand, shoulder, or arm problems (21 items). There are five alternative responses for each query. The outcomes are then used to generate a scale score that spans from zero (0) (no disability) to a hundred 100 (most severe disability) using the scores for all items.

This score can be used to measure the level of disability that individuals with hand and wrist conditions feel as well as to track changes in symptoms and function over time [20].

### Radiographic indexes

The following radiographic measurements were taken on x-rays performed 2 to 3 months after surgery:

- Radial height was calculated by measuring the length between two parallel lines drawn perpendicular to the long axis of the radial shaft, one from the ulnar corner of the lunate fossa and the other from the tip of the radial styloid, using millimeters as the unit of measurement [3].
- Ulnar variance was determined by measuring the axial length difference between the most distal extent of the ulnar head and the ulnar corner of the sigmoid notch of the radius on the PA view, using millimeters as the unit of measurement [3].
- Radial inclination was measured by calculating the angle formed by two lines: one drawn at the lunate fossa's ulnar corner perpendicular to the long axis of the radius, and the other between that point and the radial styloid's tip, using degree as the unit of measurement [3].
- Volar tilt was determined by measuring the angle formed by a line drawn from the dorsal tip to the palmar tip of the distal radius and a second line perpendicular to the long axis of the radius, using degree as the unit of measurement [3].

Fractures were classified using the Fernandez Classification [3].

### Testing for normality

The data underwent testing for normality and outliers initially. Skewness and kurtosis are statistical measures used to describe the shape and distribution of a dataset. They provide information about the deviation from symmetry and the presence of extreme values in the data set. The acceptable range for normality can vary depending on the context and the specific statistical test being used. However, there are some commonly used guidelines to assess the normality of a distribution.

For skewness, a general rule of thumb is that values between -2 and + 2 are considered approximately symmetric or normally distributed. Skewness values outside this range suggest departures from normality.

For kurtosis, a general guideline is that values around 3 (plus or minus a small margin) indicate a distribution that is similar to the normal distribution.

The results in Table 1 showed that the skewness and kurtosis of fall variables are within the acceptable range of Normality, these are: Age, Radial height, Radial inclination and Fernandez. On the other hand, three variables are not within the acceptable range, which are: Ulnar variance, Volar tilt and DASH score.

**Table 1:** Skewness and kurtosis shape and distribution

	N	Skewness		Kurtosis	
	Statistic	Statistic	Std. Error	Statistic	Std. Error
Age	140	-0.44	0.20	-0.95	0.41
Radial height	140	-0.18	0.20	0.72	0.41
Ulnar variance	140	3.03	0.20	22.70	0.41
Radial inclination	140	-0.97	0.20	3.14	0.41
Volar tilt	140	2.86	0.20	12.98	0.41
Fernandez	140	1.51	0.20	0.95	0.41
DASH score	140	3.68	0.20	16.29	0.41

### Analysis

SPSS 25 will be used to analyze the data. For normally distributed data, Pearson correlation (two-tailed) was utilized to test the bivariate correlation between each of the following: radial height, ulnar variance, radial inclination, and volar tilt with the DASH score, the Fernandez classification, and different surgical methods. T-tests were used to compare the means of DASH score means and Fernandez means by gender, ethnicity, right/left, and severity of trauma (high or low energy). An ANOVA test was used to compare the means of DASH score means and Fernandez means by surgical method. For data that is not normally distributed, Spearman correlation (two-tailed) was used to test bivariate correlation between each of the following: radial height, ulnar variance, radial inclination, and volar tilt with the DASH score, the Fernandez classification, and different surgical methods (Table 2). Mann–Whitney tests were used to compare the means of DASH score means and Fernandez means by gender, ethnicity, right/left, and severity of trauma (high or low energy). Kruskal–Wallis tests were used to compare the means of DASH score means and Fernandez means by surgical method. DASH score values were categorized into 0 and > 0, half of the sample reported a score of 0 and the other half reporting scores greater than 0, it has important implications for the statistical analysis of the data. With a balanced distribution of DASH scores, the two groups are relatively equally represented within the sample. This balance improves the statistical robustness of dichotomizing DASH scores, as it helps mitigate potential issues related to sample size imbalance, which could otherwise affect the precision and reliability of the analysis. Balanced group sizes can also increase the statistical power of analyses comparing the two groups. With a larger number of observations in each group, statistical tests are more sensitive to detecting true differences between groups, thus enhancing the likelihood of identifying significant associations or effects.

**Table 2:** Dash score’s correlation using spearman

Correlations			
			DASH score
Spearman's rho	Age	Correlation Coefficient	0.428
		*P*-value	0.000
	Radial height	Correlation Coefficient	-0.045
		*P*-value	0.598
	Ulnar variance	Correlation Coefficient	-0.254
		*P*-value	0.002
	Radial inclination	Correlation Coefficient	-0.152
		*P*-value	0.074
	Volar tilt	Correlation Coefficient	-0.082
		*P*-value	0.335
	Fernandez	Correlation Coefficient	0.254
		*P*-value	0.002

At *p*-values < 0.05, differences were regarded as significant, adhering to a widely accepted threshold in statistical analysis. This threshold is crucial as it helps control Type I error rates, ensuring that the likelihood of falsely rejecting a true null hypothesis remains low. Moreover, we recognize the potential impact of multiple testing, where conducting numerous statistical comparisons increases the likelihood of obtaining false positives by chance alone. To mitigate this risk, we employed rigorous statistical techniques to manage multiple comparisons. By implementing these strategies, we aimed to uphold the validity and reliability of our findings while minimizing the potential for spurious associations.

## Results

### Sample description

This study analyzed the outcomes of 140 patients who underwent surgery for distal radius fractures. The sample consisted of 78 females and 62 males, with a mean age of 47 years. The majority of patients had a right-sided fracture (*n* = 71) compared to left-sided (*n* = 67). The severity of trauma was categorized as low in 121 cases and high in 19 cases. The surgical method used was predominantly DVR (*n* = 70) followed by Kapandji (*n* = 45), Percutaneous pinning (*n* = 14), and closed reduction (*n* = 11).

### Dash score

**Fig. 1:**
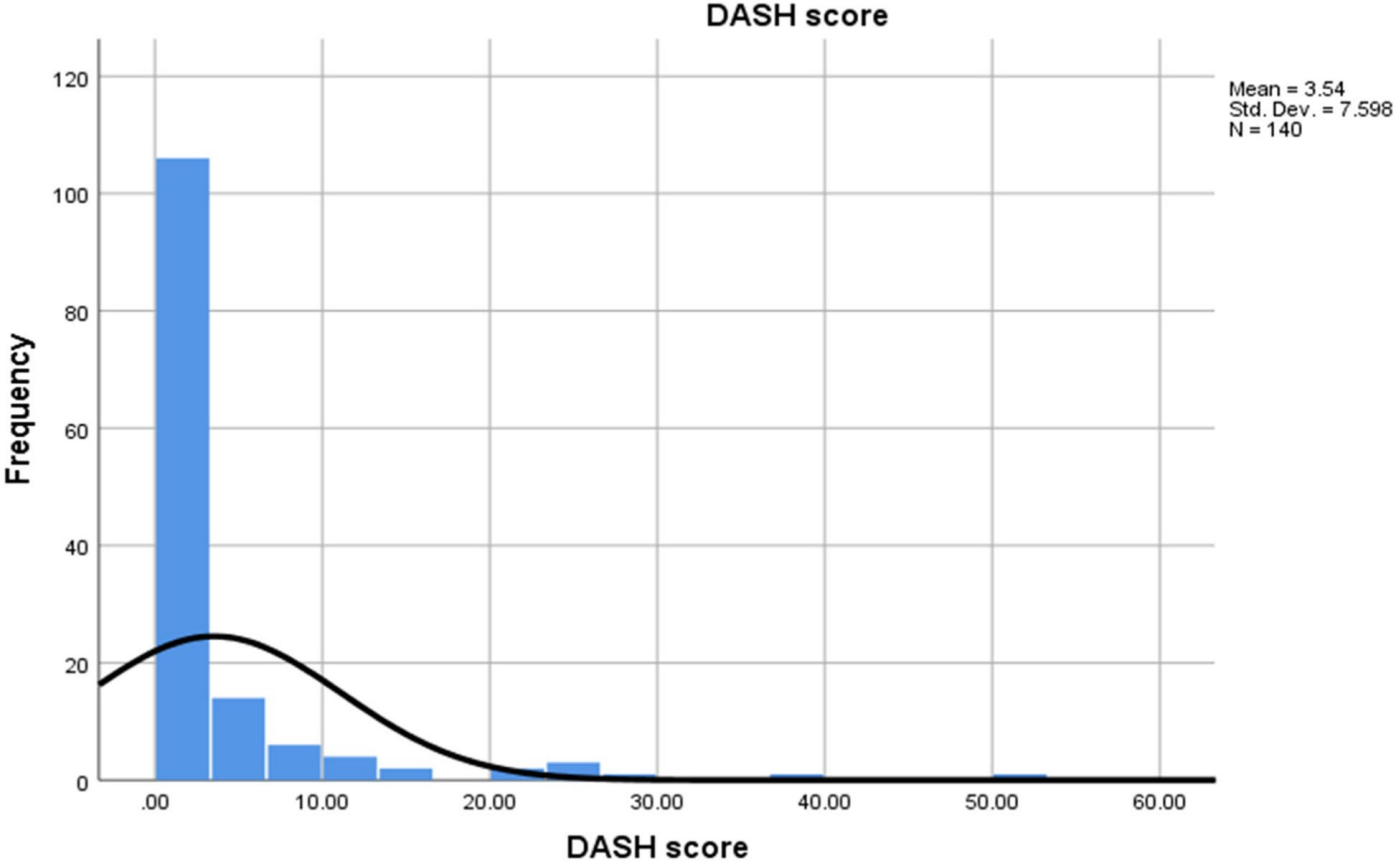
Histogram of DASH score frequency

In this study, the mean DASH score of 3.54. (Fig. 1) 66 patients scored 0 (47.1%) meaning no disability at all and 74 patients scored > 0 (52.9%) meaning presence of a disability. A DASH score of 10, is considered to reflect an important clinical change [21].

Opening a tight jar was the question with the lowest score, followed by carrying a heavy object, followed by pain on activity.

The results of the Spearman correlation test is positive between Age and DASH score, It indicate that there is a medium significant correlation (*r* = 0.43; *p* = 0), this correlation suggests that older individuals are more likely to experience higher levels of disability in their arm, shoulder, and hand function compared to younger individuals. On the other hand, we observed a negative result in the correlation test between Radial Height and the DASH score, Radial Inclination and the DASH score, and Volar Tilt and the DASH score. Specifically, we found no correlation between Radial Height and the DASH score (*p* = 0.6; *r* = -0.04), nor between Radial Inclination and the DASH score (*p* = 0.07; *r* = -0.15), or between Volar Tilt and the DASH score (*p* = 0.34;*r* = -0.08). The lack of correlation between Radial Height, Radial Inclination, Volar Tilt, and DASH scores suggest that these factors may not be strong predictors of a patient's DASH score. However, the test was positive between Ulnar variance and DASH score and Fernandez and DASH score, there was a weak significant positive correlation found between Ulnar variance and DASH score (*r* = 0.25; *p* = 0), as well as between Fernandez and DASH score (*r* = 0.25; *p* = 0). Ulnar variance and Fernandez classification may serve as a valuable clinical indicator for functional disability in patients with wrist conditions.

The Mann–Whitney test was conducted for the Right/Left wrist and DASH score, and no significant difference was found between the means (*P* = 0.41) with the following sample size: Right = 71 participants; Left = 67 participants; Right + left = 2 participants. This implies a degree of bilateral symmetry in functional impairment, indicating that both wrists are similarly affected in terms of disability as measured by the DASH score.

Additionally, the test was performed for the severity of trauma (High or Low energy) and DASH score, and the difference between the means was also not significant (*P* = 0.96), with the following sample size: High energy = 19 participants; Low energy = 121 participants. This implies that the nature or mechanism of injury may not be a strong predictor of functional outcomes in terms of wrist disability.

To assess the relationship between the Surgical method and the Dash score, we conducted the Kruskal–Wallis test and found a significant difference between the means (*p* = 0.04), with the following sample sizes: Closed reduction = 11 participants, DVR = 70 participants, Kapandji = 45 participants, interfocal pinning = 14 participants. It suggests that the choice of surgical method may influence postoperative functional outcomes, as measured by the DASH score. Overall, the significant difference in DASH scores among different surgical methods highlights the importance of carefully considering the selection of surgical approach in the management of wrist conditions. By understanding the implications of each surgical method on postoperative functional outcomes, clinicians can optimize treatment strategies and improve patient satisfaction and quality of life (Table 2).

## Discussion

Reports have shown an increase in prioritizing patient-reported disability over radiological measurement in outcome evaluation. Validated patient-rating scales have been implemented to measure disability and have demonstrated efficacy in detecting changes in outcomes [22, 23]. Although there is evidence of a favorable correlation between radiological indexes and contemporary patient-rating scales in distal radius fractures, we conducted our own study to examine the association between radiological indexes and the DASH score after such fractures [24, 25].

Several studies have suggested that achieving proper restoration of anatomy is essential for obtaining favorable outcomes following distal radius fractures [23, 26–29]. However, there is no agreement on which radiological parameter is the most reliable predictor. While some have emphasized the significance of radial shortening, others have emphasized the significance of regaining a normal palmar tilt for wrist function and carpal alignment [30–32].

In our research, we have found that ulnar variance was associated with the DASH score, whereas radial height, radial inclination, and volar tilt are not. In a cadaveric experiment, Adams shown that, in comparison to loss of radial inclination and palmar tilt, positive ulnar variance produced the most variation in the kinematics of the DRUJ and the highest distortion of the triangular fibrocartilage [33]. According to a biomechanical analysis, the distal ulna bears a much greater strain when ulnar variation is increased by 2.5 mm [15]. Prior studies have produced mixed results regarding the relationship between radiological parameters and patient-reported outcomes.

While some studies have found a link between radiological parameters and patient-reported outcomes, others have disputed this claim. Table 3 provides an overview of reported findings on this topic. To the best of our knowledge, no prior research has found a link between the validated DASH score, which measures patient-perceived disability, and all four radiological indexes.

**Table 3:** Reported results on the correlation between radiological and patient-perceived outcome after distal radius fractures

First Author	Study Design	No. of Patients	Age	Treatment	Score	Final Radiologic Parameters	Reported Results
Karnezis et al. (2005) [34]	Prospective cohort, follow-up 1 yr	30	Mean 46 (18–76) yr	Closed reduction, pinning and cast	PRWE	RS, DA, RA	Correlation between RS and PRWE and between DA and pain subscore
Gliatis et al. (2000) [35]	Retrospective, follow-up 4.9 yr	160	Median 35 (17–49) yr	Various methods of treatment	Modified PEM	RS, DA, RA, articular step/gap	Correlation between DA > 10° and subscore and between articular step and pain subscore
Chung et al. (2007) [37]	Prospective cohort, follow-up 1 yr. Smith’s fractures also included	66	Mean 49 yr	Open reduction and internal fixation	MHQ	RS, DA, RA, articular step/gap	Found correlation between articular incongruity and MHQ at 3 mo (not at 1 yr)
Kumar et al. (2007) [36]	Prospective cohort,follow-up 14 (7–34) mo	95	Mean 67 (22–94) yr	Closed reduction and cast	DASH, MHQ	RS, DA, RA	Found correlation DA < 15° and ‘‘satisfactory’’ scores in patients < 60 yr Acceptable scores despite RS > 5 mm, DA > 15°, and RA > 15° in patients > 60 yr

The findings from several referenced studies offer insights into the correlation between patient-reported outcomes and radiological indexes in wrist conditions. A lower Patient-rated Wrist Evaluation (PRWE) score was found to be correlated with increased radial shortening, according to research by Karnezis et al., indicating that deviations in radial anatomy may impact functional outcomes. This finding aligns with our study's positive correlation between ulnar variance and DASH scores, suggesting that anatomical variations can influence patient-reported disability across different assessment tools [34].

Similarly, Gliatis et al. demonstrated a relation between dorsal tilt exceeding 10 degrees and poorer subscore in a modified PEM questionnaire (Patient Evaluation Measure). While their focus was on dorsal angulation, their findings underscore the importance of radiological parameters in predicting patient-reported outcomes. This parallels our study's exploration of various radiological parameters and their association with DASH scores, emphasizing the significance of considering different anatomical aspects in assessing functional disability [35].

According to Kumar et al., there is a relationship, especially in younger patients, between appropriate dorsal tilt and favorable patient-rated outcomes as determined by the Michigan Hand Outcomes Questionnaire (MHQ) and the DASH score. This highlights the age-related nuances in the correlation between patient-reported outcomes and radiological indexes, suggesting that factors such as age may influence the impact of anatomical variations on functional disability [36].

Chung et al.'s findings regarding precise articular reduction and short-term improvements in MHQ scores after surgery further emphasize the importance of surgical interventions in optimizing patient-reported outcomes. While their study did not find significant correlations between radiological parameters and MHQ scores, it highlights the complex interplay between surgical techniques, anatomical outcomes, and patient-reported measures in determining functional disability [37].

Our study's positive correlation between inferior radiological outcomes and poorer DASH scores supports the notion that restoration of anatomy is crucial for perceived outcomes. However, it's noteworthy that, except for ulnar variance, no significant linear correlation was found between DASH scores and radiological parameters in our study. This suggests that the relationship between radiological measurements and patient-reported disability may be multifaceted, influenced by factors beyond fracture-related anatomy, such as education level and injury compensatory status, as highlighted by MacDermid et al. [38].

The positive correlation between inferior radiological outcomes and poorer DASH scores underscores the importance of addressing both anatomical abnormalities and functional disability in the management of distal radius fractures. Treatment decisions may prioritize interventions aimed at improving radiological outcomes, with consideration for tailored rehabilitation strategies to optimize functional recovery. Long-term follow-up is crucial for monitoring progression and addressing any recurrence of malunion or functional limitations, ultimately enhancing patient outcomes and quality of life.

The patient's perceived outcome may not only be correlated with radiological results, as factors like damage of the cartilage or soft-tissue injury may also have a significant impact.

Small disabilities were reported by the population under study (mean DASH score of 3.54 points). In our material, 47.1% of the patients reported having no disability at all (0 points). It is possible that the skewed distribution observed in the studied population could weaken the correlations when using the DASH score to evaluate outcomes of patients after distal radius fractures with low disability scores. This may suggest that factors beyond radiological results, such as soft-tissue injury or cartilage damage, could have a significant impact on patient perceived outcomes. Furthermore, one limitation in wrist assessment may be that the DASH score does not take into account whether the patient uses the injured or uninjured wrist for a particular job. Despite these limitations, several reports have demonstrated that the DASH score is a sensitive tool for evaluating the wrist. However, the DASH score does not focus only on the wrist; rather, it views the upper extremities as a unified functional unit [39, 40].

The moderate correlation between age and DASH score suggests that there is a relationship between these two variables in the context of wrist injuries. As age increases, there is a tendency for DASH scores to also increase, indicating poorer functional outcomes. This finding has several implications that can be considered: Age-related expectations: Older individuals may have different expectations and priorities when it comes to recovery and functional outcomes following a wrist injury compared to younger individuals. Older patients may prioritize pain management and basic activities of daily living, while younger patients may prioritize returning to work or sports activities. Understanding these age-related expectations can help healthcare providers tailor treatment plans and set realistic goals for recovery.

Functional abilities: Age-related changes in physical function, strength, flexibility, and coordination can impact the recovery process and functional outcomes following a wrist injury. Older individuals may have reduced muscle strength, joint mobility, and balance, which can affect their ability to perform daily tasks and participate in rehabilitation exercises. These age-related factors may contribute to the higher DASH scores observed in older patients.

Inherent limitations of our retrospective cohort study include the potential for selection bias due to the reliance on existing medical records and imaging reports, which may not fully capture the diversity of patients with distal radius fractures. Additionally, the retrospective nature of the study limits our ability to control for all potential confounding variables, such as comorbidities and previous injuries, which may influence the relationship between DASH scores and radiological indexes. Furthermore, while our study provides valuable insights into the correlation between these variables within our specific patient population, the findings may have limited generalizability to broader populations due to the inherent biases in data collection and patient selection methods.

Having a baseline score can be useful due to the subjectivity of patient-rated outcome surveys and the variability in disability perception. However, this retrospective study lacks a baseline DASH score, which could have minimized the impact of bias on the findings. To mitigate this risk, patients with upper extremity disabilities were excluded from the study. The absence of baseline DASH scores in this retrospective study could potentially impact the conclusions drawn from the analysis. Without baseline measures, it becomes challenging to accurately assess the change in functional outcomes over time and attribute improvements or deteriorations solely to the wrist injury. The lack of baseline data may introduce bias and confound the interpretation of results, as there is no reference point to compare the post-injury DASH scores against.

To address this limitation and enhance the robustness of future studies, researchers should consider incorporating baseline measures such as initial DASH scores at the onset of injury or treatment. By collecting baseline data, researchers can establish a more accurate baseline for each patient, enabling a more comprehensive analysis of changes in functional status over time. Including baseline measures can help control for individual differences, variability in disability perception, and potential biases that may influence the interpretation of results.

## Conclusion

This cohort study found that malunion in patients with distal radius fractures may not necessarily have higher levels of patient-rated disability, as measured by the DASH score. Nonetheless, there is a complex correlation between the patient-perceived disability and the final radiological measurements. We suggest that patient-rating scales, which may capture some features that are significant to the patient, can be helpful in assessing therapies following distal radius fractures. However, objective measurements should not be replaced by subjective scales.

While our study sheds light on the correlation between DASH scores and radiological indexes in distal radius fractures, there remain several avenues for future research to explore. Longitudinal studies assessing the trajectory of functional outcomes over time following malunited distal radius fractures could elucidate the long-term implications of radiological malunion on patient-reported disability and quality of life. Finally, comparative studies evaluating the effectiveness of various treatment approaches, including surgical interventions and conservative management, in mitigating functional impairment in patients with malunited distal radius fractures are warranted to inform evidence-based clinical practice and optimize patient outcomes.

## Data Availability

The Data is available as Excel files that I can send to you if needed. Anthony Hassoun: Anthony.j.hassoun@net.usek.edu.lb

## References

[1] Handoll HHG, Elliott J, Iheozor-Ejiofor Z, Hunter J, Karantana A. Interventions for treating wrist fractures in children. Cochrane Database Syst Rev . 2018;2018(12). Available from: https://www.ncbi.nlm.nih.gov/pmc/articles/PMC6516962/. [Cited 2022 Sep 27]. 10.1002/14651858.CD012470.pub2 PMC6516962 30566764

[2] Garfin SR, Mubarak SJ, Evans KL, Hargens AR, Akeson WH (1981). Quantification of intracompartmental pressure and volume under plaster casts. J Bone Joint Surg- Series A.

[3] Buckley R, Moran C, Apivatthakakul T (2018). AO principles of fracture management.

[4] Court-Brown C, Heckman J, McQueen M, Ricci W, Tornetta P (2014). Rockwood and Green’s Fractures in Adults.

[5] Wright NC, Hooker ER, Nielson CM, Ensrud KE, Harrison SL, Orwoll ES, et al. The epidemiology of wrist fractures in older men: The Osteoporotic Fractures in Men (MrOS) study. Osteoporos Int. 2018;29(4):859. Available from: https://www.ncbi.nlm.nih.gov/pmc/articles/PMC5939930/. [Cited 2022 Sep 25]. 10.1007/s00198-017-4349-9 PMC5939930 29344692

[6] Ng CY, McQueen MM. What are the radiological predictors of functional outcome following fractures of the distal radius? J Bone Joint Surg - Series B. 2011;93 B(2):145–50. Available from: https://online.boneandjoint.org.uk/doi/abs/10.1302/0301-620X.93B2.25631. [Cited 2022 Sep 27]. 10.1302/0301-620X.93B2.25631 21282750

[7] Mauck BM, Swigler CW (2018). Evidence-based review of distal radius fractures. Orthop Clin North Am.

[8] Jenkins NH, Mintowt-Czyz WJ (1988). Mal-union and dysfunction in Colles’ fracture. J Hand Surg Br..

[9] Wilcke MKT, Abbaszadegan H, Adolphson PY. Patient-perceived outcome after displaced distal radius fractures. A comparison between radiological parameters, objective physical variables, and the DASH score. J Hand Ther. 2007;20(4):290–9. Available from: https://pubmed.ncbi.nlm.nih.gov/17954350/. [Cited 2024 Feb 9]. 10.1197/j.jht.2007.06.001 17954350

[10] Andersen DJ, Blair WF, Steyers J, Adams BD, El-Khouri GY, Brandser EA (1996). Classification of distal radius fractures: An analysis of interobserver reliability and intraobserver reproducibility. J Hand Surg Am.

[11] Yinjie Y, Gen W, Hongbo W, Chongqing X, Fan Z, Yanqi F (2020). A retrospective evaluation of reliability and reproducibility of Arbeitsgemeinschaftfür Osteosynthesefragen classification and Fernandez classification for distal radius fracture. Medicine (United States)..

[12] Fanuele J, Koval KJ, Lurie J, Zhou W, Tosteson A, Ring D. Distal radial fracture treatment: what you get may depend on your age and address. J Bone Joint Surg Am. 2009;91(6):1313. Available from: https://www.ncbi.nlm.nih.gov/pmc/articles/PMC2686132/. [Cited 2022 Nov 7]. 10.2106/JBJS.H.00448 PMC2686132 19487507

[13] Shapiro LM, Kamal RN (2022). American Academy of Orthopaedic Surgeons appropriate use criteria: treatment of distal radius fractures. J Am Acad Orthop Surg..

[14] Mauck BM, Swigler CW (2018). Evidence-based review of distal radius fractures. Orthop Clin North Am.

[15] Canale ST, Beaty JH, Azar FM. Campbell's operative orthopaedics. 13th ed. In: Daugherty K, Jones L, editors. Elsevier; 2017.

[16] Diaz-Garcia RJ, Oda T, Shauver MJ, Chung KC (2011). A systematic review of outcomes and complications of treating unstable distal radius fractures in the elderly. J Hand Surg Am..

[17] Hess DE, Carstensen SE, Moore S, Dacus AR (2020). Smoking increases postoperative complications after distal radius fracture fixation: a review of 417 patients from a level 1 trauma center. HAND.

[18] Beumer A, Lindau TR. Grip strength ratio: a grip strength measurement that correlates well with DASH score in different hand/wrist conditions. BMC Musculoskelet Disord. 2014;15(1). Available from: https://www.ncbi.nlm.nih.gov/pmc/articles/PMC4197251/. [Cited 2022 Nov 12]. 10.1186/1471-2474-15-336 PMC4197251 25287605

[19] Development of an upper extremity outcome measure: the DASH (disabilities of the arm, shoulder and hand) [corrected]. The Upper Extremity Collaborative Group (UECG) - PubMed. Available from: https://pubmed.ncbi.nlm.nih.gov/8773720/. [Cited 2022 Nov 13]. 10.1002/(SICI)1097-0274(199606)29:6<602::AID-AJIM4>3.0.CO;2-L 8773720

[20] Gummesson C, Atroshi I, Ekdahl C (2003). The disabilities of the arm, shoulder and hand (DASH) outcome questionnaire: longitudinal construct validity and measuring self-rated health change after surgery. BMC Musculoskelet Disord..

[21] Ghassemi Jahani SA, Danielson B, Karlsson J, Danielsson AJ (2014). Long-term follow-up of thalidomide embryopathy: Malformations and development of osteoarthritis in the lower extremities and evaluation of upper extremity function. J Child Orthop.

[22] MacDermid JC, Richards RS, Donner A, Bellamy N, Roth JH (2000). Responsiveness of the short form-36, disability of the arm, shoulder, and hand questionnaire, patient-rated wrist evaluation, and physical impairment measurements in evaluating recovery after a distal radius fracture. J Hand Surg Am..

[23] Bialocerkowski AE, Grimmer KA, Bain GI (2003). Validity of the patient-focused wrist outcome instrument: do impairments represent functional ability?. Hand Clin..

[24] Karnezis IA, Panagiotopoulos E, Tyllianakis M, Megas P, Lambiris E (2005). Correlation between radiological parameters and patient-rated wrist dysfunction following fractures of the distal radius. Injury..

[25] Chung KC, Kotsis SV, Kim HM (2007). Predictors of functional outcomes after surgical treatment of distal radius fractures. J Hand Surg Am..

[26] McQueen MM (1998). Redisplaced unstable fractures of the distal radius. A randomised, prospective study of bridging versus non-bridging external fixation. J Bone Joint Surg Br..

[27] Gliatis JD, Plessas SJ, Davis TRC (2000). Outcome of distal radial fractures in young adults. J Hand Surg Br..

[28] McQueen M, Caspers J (1988). Colles fracture: does the anatomical result affect the final function?. J Bone Joint Surg Br..

[29] Solgaard S (1988). Function after distal radius fracture. Acta Orthop Scand..

[30] McQueen M, Caspers J (1988). Colles fracture: does the anatomical result affect the final function?. J Bone Joint Surg Br..

[31] Gliatis JD, Plessas SJ, Davis TRC (2000). Outcome of distal radial fractures in young adults. J Hand Surg Br..

[32] Taleisnik J, Watson HK (1984). Midcarpal instability caused by malunited fractures of the distal radius. J Hand Surg Am..

[33] Adams BD (1993). Effects of radial deformity on distal radioulnar joint mechanics. J Hand Surg..

[34] Karnezis IA, Panagiotopoulos E, Tyllianakis M, Megas P, Lambiris E (2005). Correlation between radiological parameters and patient-rated wrist dysfunction following fractures of the distal radius. Injury..

[35] Gliatis JD, Plessas SJ, Davis TRC (2000). Outcome of distal radial fractures in young adults. J Hand Surg Br..

[36] Kumar S, Penematsa S, Sadri M, Deshmukh SC (2008). Can radiological results be surrogate markers of functional outcome in distal radial extra-articular fractures?. Int Orthop..

[37] Chung KC, Kotsis SV, Kim HM (2007). Predictors of functional outcomes after surgical treatment of distal radius fractures. J Hand Surg Am..

[38] MacDermid JC, Donner A, Richards RS, Roth JH (2002). Patient versus injury factors as predictors of pain and disability six months after a distal radius fracture. J Clin Epidemiol..

[39] MacDermid JC, Richards RS, Donner A, Bellamy N, Roth JH (2000). Responsiveness of the short form-36, disability of the arm, shoulder, and hand questionnaire, patient-rated wrist evaluation, and physical impairment measurements in evaluating recovery after a distal radius fracture. J Hand Surg Am..

[40] Atroshi I, Gummesson C, Andersson B, Dahlgren E, Johansson A (2000). The disabilities of the arm, shoulder and hand (DASH) outcome questionnaire: reliability and validity of the Swedish version evaluated in 176 patients. Acta Orthop Scand..

